# Effects of limiting digital screen use on well-being, mood, and biomarkers of stress in adults

**DOI:** 10.1038/s44184-022-00015-6

**Published:** 2022-10-12

**Authors:** Jesper Pedersen, Martin Gillies Banke Rasmussen, Sarah Overgaard Sørensen, Sofie Rath Mortensen, Line Grønholt Olesen, Søren Brage, Peter Lund Kristensen, Eli Puterman, Anders Grøntved

**Affiliations:** 1 Department of Sports Science and Clinical Biomechanics, Research unit for Exercise Epidemiology, Centre of Research in Childhood Health, University of Southern Denmark, Odense, Denmark; 2 Steno Diabetes Center Odense, Odense University Hospital, Odense, Denmark; 3 Research unit PROgrez, Department of Physiotherapy and Occupational Therapy, Naestved-Slagelse-Ringsted Hospitals, Region Zealand, Denmark; 4 MRC Epidemiology Unit, University of Cambridge, Cambridge, United Kingdom; 5 Fitness, Aging, and Stress lab, School of Kinesiology, University of British Columbia, Vancouver, BC Canada

**Keywords:** Psychology, Human behaviour, Biomarkers

## Abstract

Studies have linked higher digital screen use with poorer mental health. However, there is limited experimental evidence to suggest a causal relationship. In this trial, we aimed to investigate the effects of limiting recreational digital screen use on mental well-being, mood, and biomarkers of stress in healthy young and middle-aged adults. We randomly allocated 89 families (including 164 adults) to participate in an extensive screen media reduction intervention or control. Participants in the intervention group were instructed to decrease their recreational screen use to less than 3 hours/week/person. Intervention compliance was assessed using applications and tv-monitors. Overall subjective mental well-being and mood, and collected daily biomarkers of stress (salivary cortisol and cortisone) was assessed at baseline and 2-week follow-up. Reducing recreational digital screen use resulted in significantly improved self-reported well-being and mood in adults allocated to the intervention compared to control. We observed no intervention effects for biomarkers of stress. (ClinicalTrials.gov**:** NCT04098913, 23/09/2019).

## Introduction

The proportion of adults with indicators of poor mental health (i.e., anxiety disorders, depression, and general mental well-being) has increased during the last decade in many countries^1–6^. During this same period, notable changes have occurred in digital technology and how to screen devices are used. Digital screen use has become a major part of people’s lives. Screen media devices provide endless opportunities such as checking the latest news, e-mails, scrolling through social media sites, streaming movies and series, or video calling friends, and much more. Although, each of these activities are not necessarily beneficial or harmful, the ubiquitous availability of digital devices and the high levels of engagement and social expectation to always be available may impact both physical and mental health. The worries about the potential harm continue to be debated heavily among health professionals, educators, and researchers^7–9^.

Digital screen use has been linked to lower self-reported mental health (e.g., increased levels of depression, perceived stress, and negative mood) in adults. A systematic review and meta-analysis found that high screen media use was associated with a 28% increase in the odds of depression based on data from seven longitudinal and 12 cross-sectional studies^10^. Also, a systematic review and meta-analysis of 37 cross-sectional studies reported a significant positive association between smartphone use and stress and anxiety^11^. While the findings based on observational studies may be affected by uncontrolled confounding or information bias caused by use of self-reported screen use^10,12^, another major limitation is the possibility of reverse causation—that is, that recreational screen use is increased as a consequence of mental health issues^8^. Recently, a few experimental studies have investigated the short-term effects of reducing social media use (not overall recreational screen use) on mental health^13–17^. However, evidence is still inconclusive primarily due to methodological limitations such as lack of objective assessment of intervention compliance or non-compliance to the intervention.

In addition to the impact on self-reported mental health, the use of screen media may also influence biomarkers of stress e.g., daily levels of cortisol and cortisone, which display a distinct diurnal pattern^18,19^. Cortisol secretion is regulated by the hypothalamus-pituitary-adrenal axis (HPA-axis) in response to stress^20–22^. A flatter diurnal cortisol slope has been associated with several physical and mental health outcomes e.g., obesity, depression, and externalizing symptoms^21^, while a higher cortisol awakening response has been associated with higher general life stress and prior-day feelings of sadness and being overwhelmed^23,24^. Few studies have investigated the relationship between the recreational use of screen media and biomarkers of stress^25–29^, but experimental studies are warranted.

Considering the limitations of previous observational and experimental studies, we aimed to investigate the causal relationship between recreational digital screen use (all digital screen use unrelated to work (or study) and measured outside self-reported working hours) and multiple measures of mental health in adults using data from a recent cluster randomized controlled trial (the SCREENS trial)^30,31^. Specifically, we investigated the efficacy of limiting recreational digital screen use on self-reported overall mental well-being, mood, and daily rhythms of biomarkers of stress (salivary cortisol and cortisone) in adults.

## Results

**Fig. 1:**
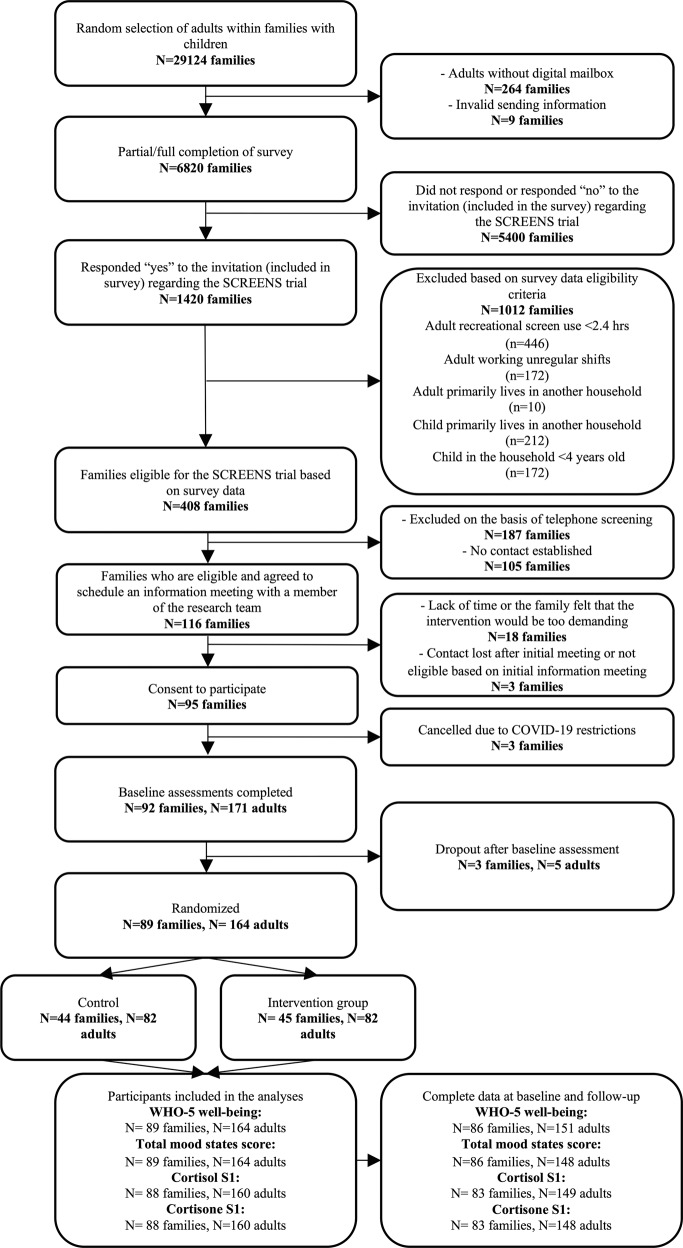
Flow of participants. Figure 1 shows a flow of participants from invitation to analyses. Families with children were recruited; this paper only reports results from adults in the families.

A total of 1420 families indicated an interest in the study and were screened for eligibility. Of these, 95 were eligible and all provided their consent to participate. A total of 92 families (including 171 adults) completed baseline measurements but three families withdrew prior to randomization. Thus, a total of 89 families (164 adults) were randomly allocated to the intervention group (45 families) or the control group (44 families) between June 2019 and March 2021 (Fig. 1).

Baseline characteristics of participants are presented for each group in Table 1. Personal factors and levels of screen media use were evenly distributed between the two groups, but participants allocated to the control group were about two years older than participants in the intervention group (Table 1).

**Table 1:** Baseline characteristics.

	Control (*n* = 44 families, *n* = 82 adults)	Intervention (*n* = 45 families, *n* = 82 adults)
**Age**		
Years, mean (sd)	42.4 (4.8)	40.2 (5.5)
**Sex**		
Female, *n* (%)	44 (54%)	44 (54%)
Male, *n* (%)	38 (46%)	38 (46%)
**Body mass index**		
Kilogram/m^2^, mean (sd)	25.8 (4.0)	26.2 (3.5)
**Educational attainment**		
ISCED 0–3, *n* (%)	16 (19%)	14 (17%)
ISCED 4–6, *n* (%)	40 (49%)	48 (59%)
ISCED 7–8, *n* (%)	26 (32%)	20 (24%)
**Individual recreational smartphone/tablet use**		
Hours/week, median (IQR)	13.8 (8.0–19.8)	14.6 (8.8–20.1)
**Individual recreational computer use**		
Hours/week, median (IQR)	2.5 (0.7–9.5)	1.2 (0.2–6.3)
**Individual recreational TV use**		
Hours/week, median (IQR)	8.8 (4.8–14.5)	8.0 (2.8–13.4)
**Household residual recreational TV use**		
Hours/week, median (IQR)	2.5 (0.5–7.7)	0.9 (0.0–5.5)
**Household recreational digital screen use**		
Hours/week, median (IQR)	96.0 (69.6–129.1)	80.3 (56.7–104.9)
**Household screen media devices**		
Devices, median (IQR)	11.0 (8.0–12.0)	9.0 (7.0–11.0)
**Children in the household**		
Number of children, median (IQR)	2.0 (2.0–3.0)	2.0 (2.0–2.0)
**Adults in the household**		
Number of adults, median (IQR)	2.0 (2.0–2.0)	2.0 (2.0–2.0)

*ISCED* International Standard Classification of Education (0–3: early childhood education to upper secondary education or equivalent, 4–6: post-secondary non-tertiary education to bachelor’s degree or equivalent, 7–8: Master’s degree to doctorate or equivalent). Household residual recreational TV use: All objectively measured TV usage that could not be assigned to individual family members.

### Recreational digital screen use during the experiment

Adults in the intervention group had a median of 2.7 h/week (IQR: 1.7 to 3.6) of recreational digital screen use, while in contrast, the control group had a median of 15.5 h/week (IQR: 9.6 to 22.8). The proportion of participants who were compliant with the intervention has been reported previously^31^.

### Well-being and mood states

All adults (control = 82, intervention = 82) were included in the analyses presented in Table 2. Participants in the intervention group significantly increased their self-reported mental well-being, whereas those in the control group did not perceive any changes in either direction. The intervention effect (mean between-group difference) for the WHO-5 Well-Being Index was 8.48 points, 95% CI: 4.90 to 12.07 (Cohen’s d: 0.72, 95% CI: 0.39 to 1.05) in favor of the screen use reduction intervention (Table 2).

**Table 2:** Well-being and mood states.

Outcomes	Control				Intervention				Intervention effect		
	Baseline		Change		Baseline		Change				
	Mean	SD	Mean change	CI	Mean	SD	Mean change	CI	Mean difference in change	CI	*P* value
**WHO-5 Well-Being Index**	65.98	13.76	1.85	−0.66 to 4.37	62.62	11.90	10.34	7.78 to 12.90	8.48	4.90 to 12.07	<0.001
**Profile of Mood States:** Total mood disturbance score	11.91	23.16	−4.63	−8.72 to −0.53	15.34	19.05	−11.46	−15.64 to −7.27	−6.83	−12.68 to −0.97	0.022
Tension score	5.49	4.07	−0.44	−1.28 to 0.40	6.18	4.07	−2.19	−3.05 to −1.34	−1.76	−2.95 to −0.56	0.004
Depression score	4.40	6.00	−1.09	−2.58 to 0.40	4.95	4.95	−2.35	−3.89 to −0.82	−1.27	−3.40 to 0.87	0.246
Anger score	6.02	4.23	−1.04	−1.94 to −0.13	5.70	3.68	−1.83	−2.76 to −0.89	−0.79	−2.09 to 0.51	0.234
Fatigue score	7.30	5.28	−0.89	−1.87 to 0.09	8.32	5.00	−2.98	−3.99 to −1.97	−2.09	−3.50 to −0.68	0.004
Confusion score	5.38	3.66	−1.14	−1.80 to −0.47	5.14	3.07	−1.41	−2.09 to −0.73	−0.27	−1.22 to 0.68	0.574
Vigor score	16.68	5.55	0.43	−0.61 to 1.47	14.94	5.15	2.31	1.25 to 3.38	1.88	0.40 to 3.37	0.013

Baseline columns present raw mean and standard deviations. Change columns present estimated within-group mean change scores and 95% confidence intervals predicted from the regression models. The mean difference in change represents the estimated intervention effect (interaction between group allocation and time adjusted for the baseline value of the outcome and age) predicted from the regression models. Linear mixed regression models were used to analyze the WHO-5 Well-Being Index and the total mood disturbance score, whereas tobit mixed regression models were used for the remaining outcomes.

Both groups significantly improved their total mood disturbance scores, but participants allocated to screen reduction had a significantly greater improvement of −6.83 points 95% CI: −12.68 to −0.97 (Cohen’s d: −0.38, 95% CI: −0.70 to −0.05) compared to control. Analyses of the mood subscales revealed significant intervention effects for tension, fatigue, and vigor in favor of the intervention (Table 2).

**Fig. 2:**
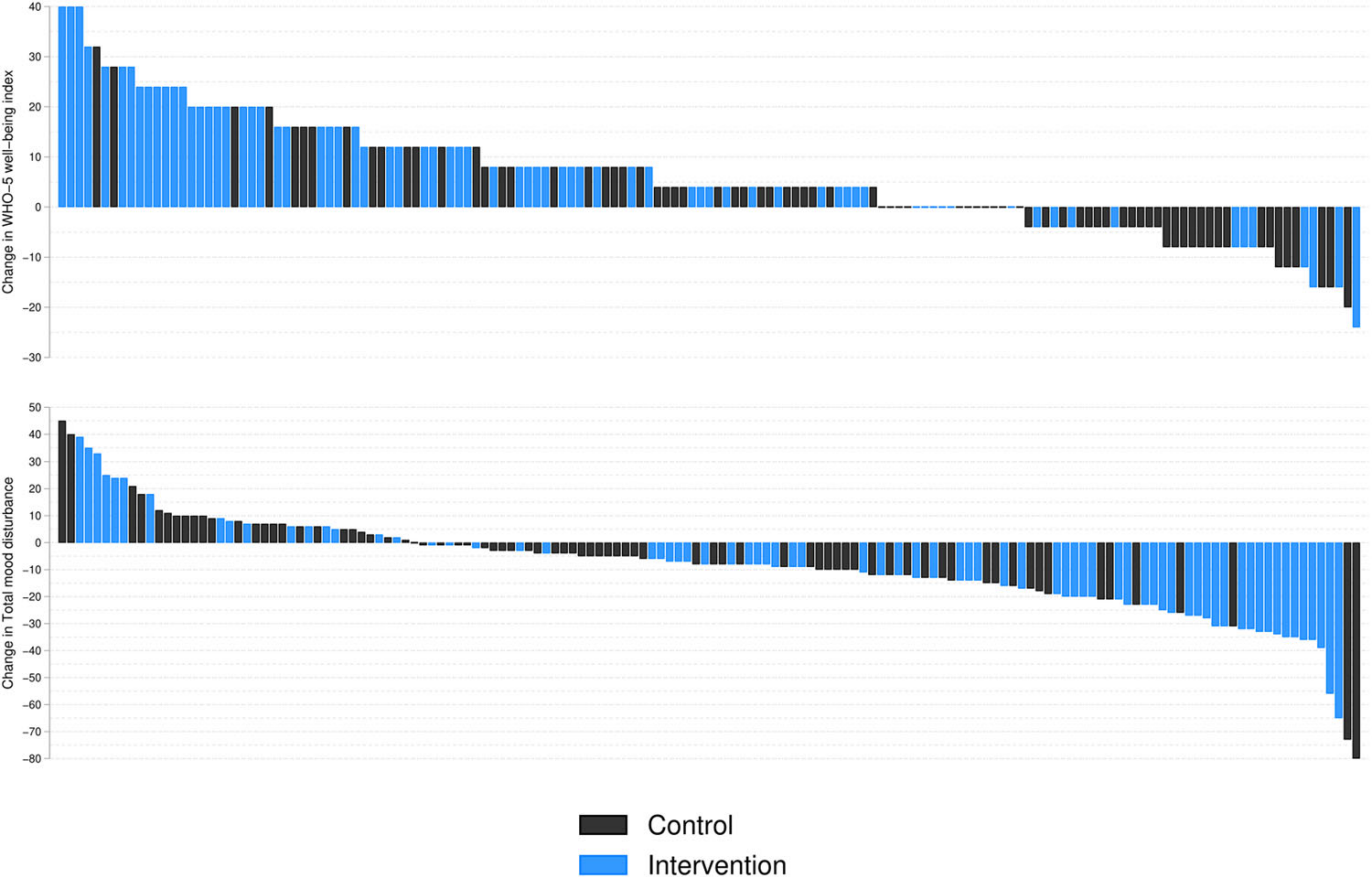
Individual participant change in WHO-5 Well-Being Index and total mood disturbance score. The figure shows individual participants unadjusted change in WHO-5 Well-Being Index (positive scores indicate improvement) and total mood disturbance (negative scores indicate improvement) for participants with complete data at baseline and follow-up (*n* = 151, 92%, and *n* = 148, 90%).

Individual participant change scores for WHO-5 Well-Being Index and total mood disturbance score are shown in Fig. 2. Raw mean scores with standard deviations of well-being and mood scores at baseline and follow-up for each group are provided in Supplementary Table 1.

### Cortisol and cortisone

**Fig. 3:**
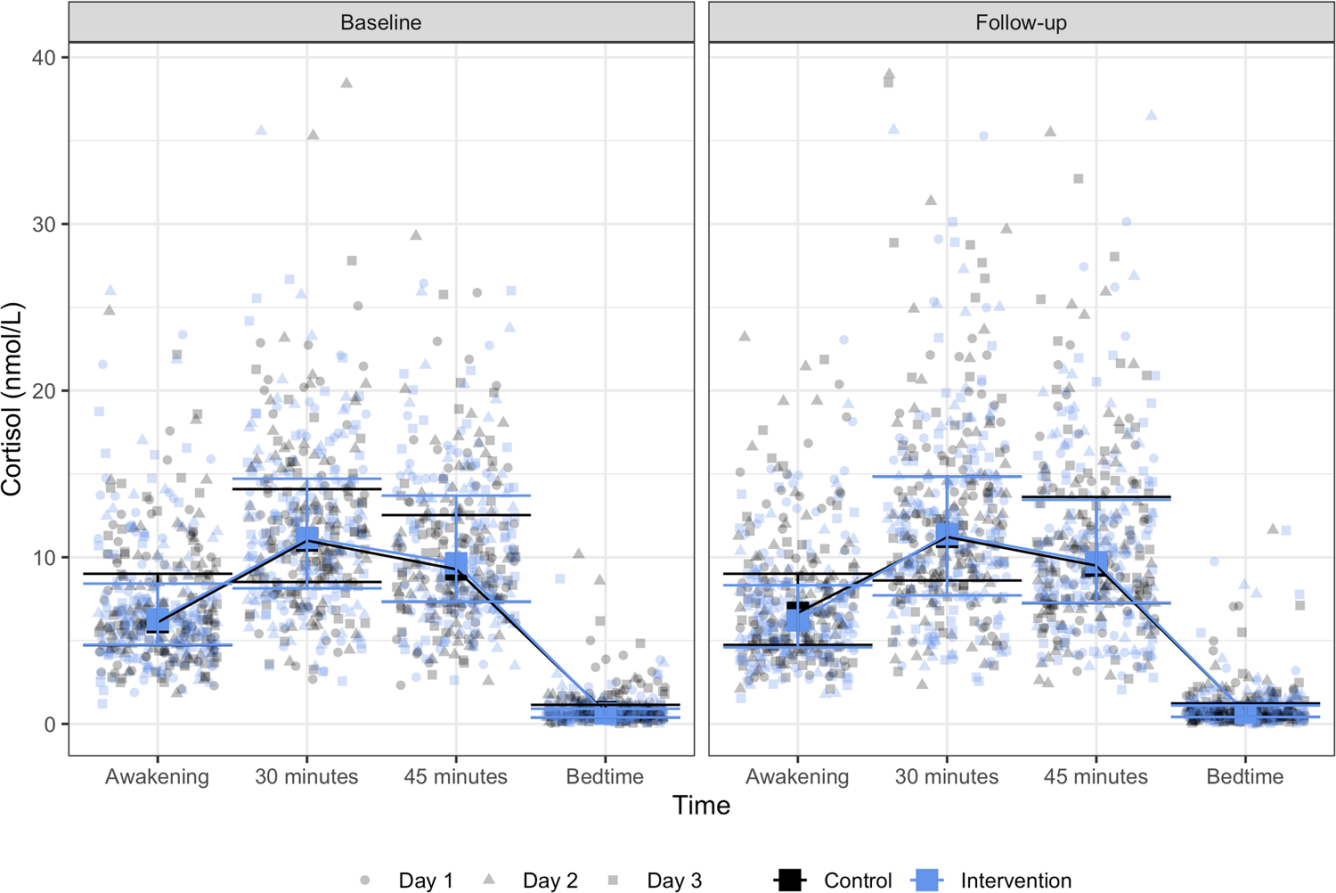
Cortisol. Figure 3 shows the 3-day median and interquartile ranges for cortisol concentration by group allocation for each time point. The figure also displays the exact cortisol concentration for each saliva sample included in the analyses after the removal of extreme outliers (points are randomly spread at each time point solely for illustration purposes).

**Fig. 4:**
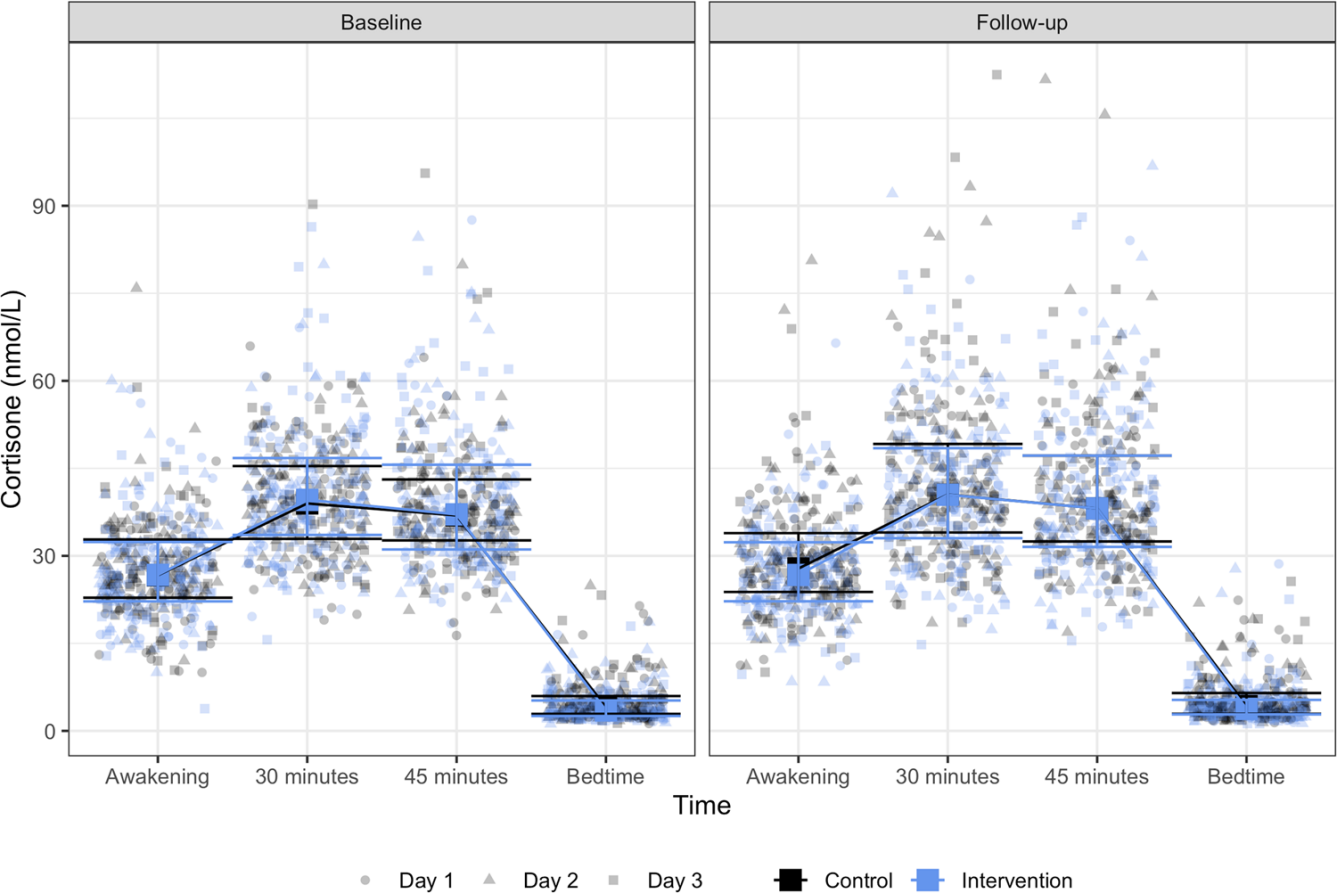
Cortisone. Figure 4 shows the 3-day median and interquartile ranges for cortisone concentration by group allocation for each time point. The figure also displays the exact cortisone concentration for each saliva sample included in the analyses after the removal of extreme outliers (points are randomly spread at each time point solely for illustration purposes).

The median number of saliva samples per participant at baseline was 11 (IQR: 9 to 12) for both control and intervention, while it was 11 (IQR: 9 to 12) for participants allocated to control and 12 (IQR: 10 to 12) for participants allocated to the intervention at follow-up. Measured cortisol and cortisone concentrations are displayed in Figs. 3, 4.

No significant within-group changes were found in any measures of daily cortisol or cortisone levels except for awakening cortisone among adults allocated to the control group (mean increase of 1.70 nmol/L (95% CI: 0.33 to 3.07)). We found no significant intervention effect on change in any of the cortisol and cortisone measures except for the awakening cortisone level (mean difference in change −1.97 nmol/L (95% CI: −3.91 to −0.03) comparing intervention vs. control) (Table 3). The standardized mean difference (Cohen’s d) for the cortisol and cortisone awakening sample were −0.08 (95%CI −0.40 to 0.22) and −0.09 (95% CI: −0.41 to 0.21). Raw mean scores with standard deviations of cortisol and cortisone measures at baseline and follow-up for each group are provided in Supplementary Table 2.

**Table 3:** Cortisol and cortisone.

Outcomes	Control (*n* = 82)				Intervention (*n* = 82)				Intervention effect		
	Baseline		Change		Baseline		Change				
	Mean	SD	Mean Change	95% CI	Mean	SD	Mean Change	95% CI	Mean difference in change	95%CI	*P* value
**Cortisol**											
Awakening sample, nmol/L	7.69	5.71	0.39	−0.22 to 0.99	7.74	5.84	−0.24	−0.85 to 0.36	−0.63	−1.48 to 0.23	0.151
Diurnal cortisol slope, nmol/L/time	−0.39	0.24	−0.00	−0.05 to 0.04	−0.41	0.27	0.02	−0.02 to 0.07	0.03	−0.04 to 0.09	0.396
Cortisol awakening response 30, nmol/L	4.66	4.98	0.16	−0.71 to 1.03	4.52	5.29	0.49	−0.37 to 1.35	0.34	−0.89 to 1.56	0.592
Cortisol awakening response 45, nmol/L	2.91	4.86	0.49	−0.43 to 1.41	3.32	5.64	0.44	−0.47 to 1.35	−0.05	−1.34 to 1.25	0.942
Cortisol awakening response peak, nmol/L	5.04	5.19	0.17	−0.70 to 1.04	5.18	5.54	0.13	−0.73 to 1.00	−0.04	−1.26 to 1.19	0.954
Cortisol awakening response *auc*_*G*_	13.82	5.06	0.27	−0.56 to 1.09	13.81	5.38	−0.01	−0.82 to 0.81	−0.27	−1.43 to 0.89	0.647
Cortisol awakening response *auc*_*I*_	2.34	5.57	0.25	−0.76 to 1.26	2.37	6.01	0.63	−0.38 to 1.63	0.38	−1.05 to 1.80	0.604
**Cortisone**											
Awakening sample, nmol/L	27.86	16.07	1.70	0.33 to 3.07	28.56	17.42	−0.27	−1.64 to 1.10	−1.97	−3.91 to −0.03	0.046
Diurnal cortisone slope, *nmol/L/time*	−1.41	0.55	−0.02	−0.13 to 0.08	−1.46	0.62	−0.02	−0.12 to 0.08	0.00	−0.14 to 0.15	0.960
Cortisone awakening response 30, nmol/L	12.33	9.41	0.91	−0.86 to 2.67	13.03	11.59	0.89	−0.87 to 2.64	−0.02	−2.51 to 2.47	0.988
Cortisone awakening response 45, nmol/L	10.37	10.60	1.18	−0.82 to 3.17	12.17	14.35	0.92	−1.05 to 2.89	−0.26	−3.06 to 2.55	0.858
Cortisone awakening response peak, nmol/L	13.86	10.18	1.28	−0.57 to 3.14	15.53	13.42	0.22	−1.62 to 2.06	−1.06	−3.67 to 1.54	0.424
Cortisone awakening response *auc*_*G*_	48.46	11.50	1.97	−0.05 to 4.00	49.65	12.13	0.81	−1.20 to 2.83	−1.16	−4.02 to 1.70	0.426
Cortisone awakening response *auc*_*I*_	5.17	10.78	0.58	−1.44 to 2.59	6.04	13.56	1.28	−0.72 to 3.29	0.71	−2.13 to 3.55	0.625

Baseline columns report raw mean and standard deviations. Change columns report estimated within-group change scores and 95% confidence intervals. Mean difference in change column report estimated intervention effects (interaction between time and group adjusted for the baseline value of the outcome and age). The number of families/participants included in the models ranged from 87–89 families/156–160 adults. See methods section “Cortisol and cortisone” for the elaboration of different cortisol and cortisone awakening response measures.

## Discussion

In this cluster randomized controlled trial, we investigated the efficacy of reducing household recreational digital screen use on overall mental well-being, mood, and daily biomarkers of stress in adults. We found significant improvements in overall self-reported mental well-being and mood in favor of recreational screen reduction. No consistent intervention effects were found for measures of daily cortisol and cortisone levels.

Our study provides novel experimental evidence that restricting recreational digital screen use increases self-reported overall mental well-being and mood. These findings are in line with results from observational studies reported in systematic reviews^10,11^. To the best of our knowledge, no other trials have investigated the impact of limiting overall recreational digital screen use on perceived mental well-being and mood. Some trials have investigated the short-term effects of limiting social media engagement on mental well-being and shown mixed results^13–17^. A key difference between previous trials and our trial is the high level of intervention compliance observed in our trial. All previous trials except for Wezel et al. used self-reported measures of compliance, which may be prone to bias^12^. Wezel et al. used an objective measure to document compliance to change in the amount of social media use and they reported no effect on well-being. However, their attempt to reduce social media use to 50 and 10% among participants in the two respective intervention groups was unsuccessful, which could explain the absence of an effect on the mental well-being outcomes. In addition, interventions in previous trials were aimed at individual participants rather than families. There are pros and cons of the individual-centered intervention design, but a substantial proportion of screen media use in families occur as part of a wider social context. Our family-based design could be an important factor for the observed effect on mental well-being and mood in our trial because it may increase intervention compliance through social change processes that can initiate and consolidate personal change^32^.

The intervention effect of 8.48 points (95% CI: 4.90 to 12.07) on the WHO-5 Well-Being Index corresponds to a standardized mean difference of 0.72 (Cohen’s d). The moderate to large standardized mean difference is noteworthy given that the population under study were healthy adults. In addition, the confidence interval overlaps 10, which is considered to be the minimal clinically important difference^33,34^. The intervention effect for the total mood disturbance score was low to moderate with a standardized mean difference of 0.38 (Cohen’s d). In relation to the results for mental well-being, it suggests that screen use has a smaller influence on mood states than on overall mental well-being. The use of screen media devices may affect well-being and mood through several pathways. One pathway may be that most adults always carry a smartphone around. The constant availability has been suggested to induce a perceived obligation to be accessible at all times, a notion that has been suggested to induce feelings of stress, depression, and guilt^35^. Another pathway may be that passive social media use affects well-being negatively through social comparison^36^. Finally, Afifi et al. suggest that engagement with screen media devices may possibly disrupt and displace fundamental behaviors such as eating, exercising, and sleeping, which may induce stress^29^.

This is the first trial exploring the effect of limiting screen media use on daily levels of cortisol and cortisone. Our study provided no consistent evidence for a causal relationship between restricting screen media use and change in biomarkers for stress, at least in the short term of 2 weeks. The absence of an effect on cortisol and cortisone may be due to a lack of statistical power to detect small effect sizes. An alternative explanation could be that our sample consisted of healthy adults. We cannot rule out that limiting screen use in adults with symptoms of stress or depression, who often display altered diurnal cortisol slopes and cortisol awakening responses^37,38^, could have a positive effect on their cortisol and cortisone profiles.

A key strength of our study is the experimental design, which increases the confidence that the observed effects of limiting screen media use on well-being and mood are causal because known and unknown confounding factors are expected to be equally distributed between the groups being compared. Another strength of our study is the implementation of objective assessments of intervention compliance^31^. Furthermore, the included participants and non-eligible participants had similar background characteristics i.e., age, sex, educational attainment, and recreational screen media use^31^. Also, results were robust after stratification for whether participants were enrolled before or after COVID-19 i.e., March 2020 (data not shown). However, the results should be interpreted with the following limitations in mind. First, due to the behavioral nature of the intervention, blinding was not possible, which may have introduced bias in the self-reported outcomes because participants may potentially have been influenced by knowledge of being in the screen use reduction group. We expect that this possible bias, to some degree, could have exaggerated the effect of screen use reduction on subjective mental well-being and mood as some participants may have responded in the direction of a hypothesis of benefit or that they think the researcher's desire (the concept of demand characteristics*)*^39^. Second, although participants in the intervention group successfully decreased their screen media use, we also observed a modest decrease in screen media use in the control group. This was likely because some families were motivated to try decreasing their screen media engagement prior to randomization. Third, although our assessment followed standard recommendations for assessment of salivary cortisol^21,22,40^, objective assessment of the timing of samples may have further strengthened our results. Finally, findings may not be generalizable beyond healthy adults who live in households with children.

Collectively, our study provides experimental evidence that limiting recreational digital screen use positively affects mental well-being and mood in adults. Our findings highlight the importance of awareness of the amount of time adults spend using recreational digital screen media devices. Future experimental studies should explore if the observed relationships depend on specific types of screen media content or different motivations for digital screen use. Furthermore, studies examining the impact of long-term reductions in screen use are warranted.

## Methods

### Study design

This study is a secondary analysis of the SCREENS trial (a parallel cluster randomized controlled trial), and it is reported in compliance with the CONSORT statement^41^. The trial design is described in detail in the study protocol^30^. The SCREENS trial was designed to investigate the efficacy of reducing household screen media use on several outcomes, and not to evaluate the pragmatic effectiveness of the intervention. We randomly allocated families (cluster unit) to reduce recreational screen use for a period of 2 weeks (intervention) or continue using screen media as usual (control). The cluster design was chosen to enhance compliance with the screen reduction intervention. The first family was enrolled on June 6, 2019 and the last family completed a follow-up on March 30, 2021.

We obtained ethical approval from the Ethical Committee of Southern Denmark (S-20170213). All participants gave written informed consent before baseline. The trial was registered at ClinicalTrials.gov **(**NCT04098913).

### Study participants

Families residing in the Region of Southern Denmark with at least one child aged 6–10 years were recruited through a population-based survey^42^. A digital survey invitation was sent to a randomly chosen adult in each family via a mandatory digital mailbox (e-Boks). The Danish Health Data Authority performed the random selection of survey invitees using data from the Danish Civil Registration System^43^. The survey included questions on the families’ screen media habits^44^. Respondents were asked to answer a question (yes/no) on whether they were interested in participating in the SCREENS trial. We assessed whether the families were eligible based on survey data using the following criteria: The responding parent had self-reported recreational screen use >40th percentile (2.4 h/day) based on the first 1000 survey responses, all children in the household had to be >4 years of age and adults had to be full-time students or employed full-time (with no regular night shifts). The responding parent from families fulfilling these criteria were telephoned to confirm that at least one adult and one child in the household would be willing to participate in the trial, and that at least one participating adult and all participating children would be able to handover their smartphone(s) and tablet(s) for a period of 2 weeks.

We excluded individual participants from eligible families if the participants were not able to engage in everyday physical activities, if they had been diagnosed with a sleep disorder, a neuropsychiatric disorder, or a developmental disorder, or if they had been on stress-related sick leave within the last 3 months.

### Randomization

The Odense Patient data Explorative Network (OPEN) generated the block randomization sequence using permuted blocks of two to four families. OPEN had no role in the delivery of the intervention or collection of data. The randomization was performed by a member of the research team via an online randomization website within the families’ households after completion of the baseline assessments. The member of the research team had no knowledge of the group allocation until after the randomization was performed. The study was open-label because blinding of participants was not achievable due to the behavioral nature of the intervention.

### Interventions

Families allocated to the control group were instructed to carry on with their usual screen media habits. Families allocated to the screen reduction intervention were instructed to change their screen media habits substantially. First, members of families assigned to the intervention group were instructed to reduce their individual recreational digital screen use to a maximum of 3 h/week during the 2-week intervention period. Second, all participants were instructed to handover their tablet(s) and/or smartphone(s) in exchange for a non-smart cell phone (Nokia 130). Some adults were not able to because they had mixed work and leisure smartphones, but at least one adult had to if the family wanted to participate. Third, families were encouraged to talk about their expected challenges of reducing recreational screen use for 2 weeks and list potential solutions. Fourth, adults were allowed up to 30 min/day of so-called necessary screen media use (e.g., arranging appointments, checking online banking, etc.). Children who had to use screens for homework were allowed to do this to the extend necessary. During the intervention, all screen media use had to be registered in simple daily diaries. Also, three to five intervention reminders were positioned in places where families gather and in rooms where family members typically use screen media. Families who completed the SCREENS experiment received a financial reimbursement of 70 Euros. The intervention components are described in more detail in the study protocol^30^.

### Assessment of intervention compliance

Recreational use of smartphones, tablets, and computers was objectively assessed using non-commercial Device Tracker apps and television use was assessed using a monitor developed in-house^31,45^. A total of 78 (95%) adults allocated to the intervention group were considered compliant with the intervention because they had less than 7 hours/week of recreational screen use during the experiment^31^. In this paper, we report descriptive statistics (median and interquartile ranges) of recreational digital screen use during the experiment period to allow for a comparison of the contrast in screen exposure.

### Outcomes

All outcomes of the SCREENS trial are described in the study protocol^30^. The following sections describe the outcomes of the current paper.

### Well-being

A digital version of the WHO-5 Well-Being Index was used to assess overall mental well-being before the baseline protocol started and immediately after the completion of the experiment period. The five-item questionnaire is widely used as an outcome measure of mental well-being in clinical trials and has acceptable validity, and is psychometrically sound^34,46^. The WHO-5 Well-Being Index consists of five statements about how a person has felt (e.g., “I have felt cheerful in good spirits”) over the previous 2 weeks. Participants had to provide one of the following answers: *All of the time (5), most of the time (4), more than half of the time (3), less than half of the time (2), some of the time (1), at no time (0)*. The final score was calculated for each adult by adding the scores from the five items and multiplying the raw sum by 4^34^. Higher scores correspond to better mental well-being.

### Mood states

Overall mood states were assessed using the Profile of Mood States questionnaire, which is a validated and widely used scale to assess mood states in healthy populations^47,48^. The questionnaire consists of 65 words (e.g., friendly, tense, angry, etc.) and participants had to answer one of the following: *Not at all (0)*, *A little (1)*, *Moderately (2)*, *Quite a lot (3)*, *Extremely (4)*. For two of the questions, the scoring was reversed (from 4 to 0). For participants who had missing data on less than 5 single items (baseline *n* = 14, follow-up *n* = 15), we imputed the observed median for each item from participants with complete data for the item. Scores were summed in six distinct subscales (tension, depression, anger, fatigue, confusion, and vigor). Total mood disturbance was calculated by adding tension, depression, anger, fatigue, and confusion scores and subtracting the vigor score^47^. Lower scores correspond to a better mood for all subscales (except for the vigor score) and total mood disturbance.

### Cortisol and cortisone

We assessed salivary biomarkers of stress (cortisol/cortisone). We used an ambulatory assessment of cortisol and cortisone to provide a more ecologically valid picture of the potential changes in daily rhythms of cortisol/cortisone secretion in response to the intervention^40^. Participants received face-to-face instructions from a member of the research team on how to complete the saliva sampling using Salivettes and synthetic swaps (Starstedt, Nümbrecht, Germany). We emphasized the importance of reporting the actual time of each sample in the daily sampling diary. Participants were also provided with a written sampling manual, including pictures. Salivary samples had to be collected on the three days leading up to randomization and during the last three days of the experiment (Supplementary Fig. 1) to increase the reliability of the measurements^49^. Participants were instructed to complete three samples in the morning (upon awakening, +30 min, +45 min) which is the minimum protocol to assess the cortisol/cortisone awakening response suggested in previous expert consensus guidelines^22^. After collecting the awakening sample, participants were instructed to start a pre-programmed alarm clock (Dual Digital Timer, S. Brannan Sons Ltd., England) for reminders for the +30 min, +45 min sample collections. Participants were also instructed to collect one sample prior to bedtime. The research team carefully instructed participants to refrain from smoking, exercising, toothbrushing, eating, or drinking anything but water during the morning sampling routine and 30 min prior to collection of the bedtime sample. Participants were instructed to place the samples in a sample rack in their freezer during the course of the study. All samples were transported in refrigerated boxes and stored in a freezer at the University of Southern Denmark. Finally, we transported the saliva samples to the clinical biochemistry department at Slagelse Hospital, Region Zealand, where the samples were analyzed. Cortisol and cortisone levels were determined using isotope dilution liquid chromatography-tandem mass spectrometry (LC-MS/MS).

We calculated daily diurnal cortisol and cortisone slopes by subtracting the bedtime concentration from the awakening concentration and dividing it by the time elapsed between the two samples^50^. The following daily cortisol and cortisone awakening response metrics were calculated: CAR30 (30 min concentration–awakening concentration), CAR45 (45 min concentration–awakening concentration), CARpeak (peak concentration at 30 or 45 min—awakening concentration), area under the curve ground (CARauc_G_), and area under the curve with respect to increase (CARauc_I_) was calculated using the two formulas provided by Pruessner et al.^51^.

Cortisol and cortisone data were positively skewed at each time point. Thus, data were log-transformed at each time point to identify extreme outliers (>±3 SD) for the cortisol or cortisone concentrations at each time point. After a case-by-case evaluation, 36 cortisol and 38 cortisone samples of the total 3636 and 3642 samples were identified as extreme outliers, respectively, and these were deleted^40^. Log-transformed variables were only used to identify extreme outliers and were not included in any analyses. Morning saliva samples and evening saliva samples deviating more than 15 min (*n* = 261) and 60 min (*n* = 118) from the planned sampling protocol, respectively, were excluded from the analyses.

### Sample size

The determination of the sample size of the SCREENS trial was based on results from our pilot study^52^, and the SCREENS trial aimed to have 80% power to detect a 24 min/day group-mean difference in children’s non-sedentary time (primary outcome in the SCREENS trial). Thus, the sample size was not powered to detect a specific change in overall well-being, mood, or daily cortisol and cortisone measures. However, results from our pilot study suggest that our total sample size of 164 adults has the power to detect standardized effect sizes >0.35 (Cohen’s d) for the cortisol and cortisone area under the curve measures^49^.

### Statistical approach

Data were analyzed using linear mixed-effects models, including an interaction term between group allocation and the baseline/follow-up variable. Mixed-effects tobit regression was used for the moods subscales to account for potential floor and ceiling effects^53^, and values were censored at 0 (lower limit) for the tension, depression, anger, fatigue, and confusion subscales, and at 32 (upper limit) for the vigor subscale. All analyses included family-level and participant-level random intercepts to account for potential correlation due to clustering. Distinct models were run with the following variables as outcomes: WHO-5 Well-Being Index, total mood disturbance score, each of the Profile of mood states subscales, cortisol and cortisone awakening sample, diurnal cortisol and cortisone slopes, and five different measures of the cortisol and cortisone awakening response (CAR30, CAR45, CARpeak, CARauc_G_, and CARauc_I_). All models were adjusted for age because there was a significant difference between the two groups at baseline. In addition to family-level and participant-level random intercepts, models for cortisol and cortisone also included a day of assessment level random intercept, because days were clustered within participants, and participants were clustered within families. We report mean baseline levels of outcomes, estimated within-group mean change in outcomes, and the intervention effect, which is the interaction between the group and the time variable from each of the linear or tobit mixed-effects models. All estimates were provided with 95% confidence intervals and analyses were conducted according to the intention-to-treat principles. We calculated Cohen’s d using the changes scores and standard deviations, and the number of participants in each group.

We found no violations of the assumptions of linear or tobit mixed-effects models. Statistical analyses were performed using STATA 17 using an α-level of 0.05 (two-sided).

### Supplementary information

Supplementary Information

## Data Availability

Deidentified data can be made available upon request for research purposes after a data handling agreement is made in accordance with the General Data Protection Regulation and the Danish Data Protection Act. Contact the corresponding author in case of data inquiries.
