# Supplementary material for: Effects of limiting digital screen use on well-being, mood, and biomarkers of stress in adults
Source: Npj Ment Health Res. 2022 Oct 12;1:14. doi: 10.1038/s44184-022-00015-6 (PMC9554843; doi:10.1038/s44184-022-00015-6)
Supplement: Supplementary file 1 — Supplementary Information [file 44184_2022_15_MOESM1_ESM.docx]

**Supplementary material**

**Effects of limiting digital screen use on mental well-being, mood, and biomarkers of stress in adults**

Jesper Pedersen^1^; Martin Gillies Banke Rasmussen^1,2^; Sarah Overgaard Sørensen^1^; Sofie Rath Mortensen^1,3^; Line Grønholt Olesen^1^; Søren Brage^1,4^; Peter Lund Kristensen^1^; Eli Puterman^5^ Anders Grøntved^1^

^1^Department of Sports Science and Clinical Biomechanics, Research unit for Exercise Epidemiology, Centre of Research in Childhood Health, University of Southern Denmark, Odense, Denmark.

^2^Steno Diabetes Center Odense, Odense University Hospital, Odense, Denmark

^3^Research unit PROgrez, Department of Physiotherapy and Occupational Therapy, Naestved-Slagelse-Ringsted Hospitals, Region Zealand, Denmark.

^4^MRC Epidemiology Unit, University of Cambridge, Cambridge, United Kingdom.

^5^Fitness, Aging, and Stress lab, School of Kinesiology, University of British Columbia, Vancouver, BC, Canada.

**Table of contents**

[Supplementary Table S1: WHO-5 Well-being Index and Mood scores 2](#_Toc112340995)

[Supplementary Table S2: Cortisol and Cortisone 3](#_Toc112340996)

[Supplementary Figure S1: Overview of salivary samples and questionnaire administration 4](#_Toc112340997)

# Supplementary Table S1: WHO-5 Well-being Index and Mood scores

| **Outcomes** | **Control (n=82)** | | | | | | **Intervention (n=82)** | | | | | |
| --- | --- | --- | --- | --- | --- | --- | --- | --- | --- | --- | --- | --- |
|  | Baseline | | | Follow-up | | | Baseline | | | Follow-up | | |
|  | n | Mean | SD | n | Mean | SD | n | Mean | SD | n | Mean | SD |
| **WHO-5 Well-being Index** | 81 | 65.98 | 13.76 | 78 | 67.64 | 12.55 | 81 | 62.62 | 11.90 | 75 | 73.17 | 11.91 |
| **Total Mood Disturbance** | 81 | 11.91 | 23.16 | 77 | 7.53 | 20.02 | 79 | 15.34 | 19.05 | 75 | 4.23 | 21.91 |
| *Tension score* | 81 | 5.49 | 4.07 | 77 | 5.12 | 3.73 | 79 | 6.18 | 4.07 | 75 | 4.12 | 3.19 |
| *Depression score* | 81 | 4.40 | 6.00 | 77 | 3.66 | 4.47 | 79 | 4.95 | 4.95 | 75 | 3.77 | 5.95 |
| *Anger score* | 81 | 6.02 | 4.23 | 77 | 4.97 | 3.66 | 79 | 5.70 | 3.68 | 75 | 4.09 | 4.52 |
| *Fatigue score* | 81 | 7.30 | 5.28 | 77 | 6.51 | 4.43 | 79 | 8.32 | 5.00 | 75 | 5.59 | 4.45 |
| *Confusion score* | 81 | 5.38 | 3.66 | 77 | 4.34 | 2.59 | 79 | 5.14 | 3.07 | 75 | 3.91 | 3.15 |
| *Vigor score* | 81 | 16.68 | 5.55 | 77 | 17.06 | 6.02 | 79 | 14.94 | 5.15 | 75 | 17.25 | 5.43 |

Supplementary Table S1 shows number of observations (participants), mean, and standard deviations at baseline and follow-up, respectively.

# Supplementary Table S2: Cortisol and Cortisone

| **Outcomes** | **Control (n=82)** | | | | | | **Intervention (n=82)** | | | | | |
| --- | --- | --- | --- | --- | --- | --- | --- | --- | --- | --- | --- | --- |
|  | Baseline | | | Follow-up | | | Baseline | | | Follow-up | | |
|  | n | Mean | SD | n | Mean | SD | n | Mean | SD | n | Mean | SD |
| **Cortisol** |  |  |  |  |  |  |  |  |  |  |  |  |
| *Awakening sample, nmol/L* | 793 | 7.69 | 5.71 | 821 | 8.09 | 6.31 | 820 | 7.74 | 5.84 | 809 | 7.74 | 6.17 |
| *Diurnal cortisol slope, nmol/L/time* | 175 | -0.39 | 0.24 | 174 | -0.39 | 0.22 | 181 | -0.41 | 0.27 | 180 | -0.39 | 0.24 |
| *Cortisol Awakening Response 30, nmol/L* | 192 | 4.66 | 4.98 | 198 | 4.86 | 5.42 | 203 | 4.52 | 5.29 | 194 | 5.05 | 5.63 |
| *Cortisol Awakening Response 45, nmol/L* | 182 | 2.91 | 4.86 | 189 | 3.42 | 5.23 | 191 | 3.32 | 5.64 | 187 | 3.85 | 5.81 |
| *Cortisol Awakening Response peak, nmol/L* | 197 | 5.04 | 5.19 | 200 | 5.23 | 5.61 | 206 | 5.18 | 5.54 | 200 | 5.48 | 5.89 |
| *Cortisol Awakening Response auc_G_* | 181 | 13.82 | 5.06 | 189 | 14.01 | 6.02 | 189 | 13.81 | 5.38 | 185 | 13.79 | 6.11 |
| *Cortisol Awakening Response auc_I_* | 181 | 2.34 | 5.57 | 189 | 2.56 | 5.44 | 189 | 2.37 | 6.01 | 185 | 3.05 | 6.07 |
| **Cortisone** |  |  |  |  |  |  |  |  |  |  |  |  |
| *Awakening sample, nmol/L* | 799 | 27.86 | 16.07 | 819 | 30.06 | 18.41 | 817 | 28.56 | 17.42 | 811 | 28.61 | 18.05 |
| *Diurnal cortisone slope, nmol/L/time* | 180 | -1.41 | 0.55 | 174 | -1.43 | 0.63 | 182 | -1.46 | 0.62 | 183 | -1.45 | 0.55 |
| *Cortisone Awakening Response 30, nmol/L* | 193 | 12.33 | 9.41 | 198 | 13.71 | 11.06 | 202 | 13.03 | 11.59 | 193 | 14.04 | 12.40 |
| *Cortisone Awakening Response 45, nmol/L* | 183 | 10.37 | 10.60 | 188 | 11.83 | 12.34 | 189 | 12.17 | 14.35 | 186 | 13.28 | 14.65 |
| *Cortisone Awakening Response peak, nmol/L* | 196 | 13.86 | 10.18 | 200 | 15.41 | 12.05 | 205 | 15.53 | 13.42 | 198 | 16.04 | 14.11 |
| *Cortisone Awakening Response auc_G_* | 182 | 48.46 | 11.50 | 188 | 50.64 | 14.76 | 186 | 49.65 | 12.13 | 184 | 50.49 | 14.05 |
| *Cortisone Awakening Response auc_I_* | 182 | 5.17 | 10.78 | 188 | 5.93 | 11.82 | 186 | 6.04 | 13.56 | 184 | 7.61 | 13.97 |

Supplementary Table S2 shows number of salivary samples (n), mean, and standard deviations at baseline and follow-up, respectively. See methods section “Cortisol and cortisone” for elaboration of different cortisol and cortisone awakening response measures.

Supplementary Figure S1: Overview of salivary samples and questionnaire administration


Supplementary Figure S1 shows a detailed description of the timing of the saliva samples and administration of the questionnaires (WHO-5 Well-being Index and Profile of Mood States) during the SCREENS trial.
